# Sexual health of young people with perinatal HIV and HIV negative young people in England

**DOI:** 10.1371/journal.pone.0205597

**Published:** 2018-10-12

**Authors:** Ali Judd, Caroline Foster, Lindsay C. Thompson, Kate Sturgeon, Marthe Le Prevost, Eva Jungmann, Katie Rowson, Hannah Castro, Diana M. Gibb, on behalf of the Adolescents and Adults Living with Perinatal HIV (AALPHI) Steering Committee

**Affiliations:** 1 MRC Clinical Trials Unit at UCL, University College London, London, United Kingdom; 2 Imperial College Healthcare NHS Trust, London, United Kingdom; 3 Central and North West London NHS Foundation Trust, London, United Kingdom; Makerere University School of Public Health, UGANDA

## Abstract

As adolescents with perinatal HIV (PHIV) survive into adulthood, gaining insight into sexual behaviour and risk-taking is important. Between 2013–2015, 296 PHIV aged 13–21 years and 96 HIV negative affected adolescents (13–23 years) were recruited to the Adolescents and Adults Living with Perinatal HIV (AALPHI) cohort in England. Sexual health data were collected through computer-assisted self-interview questionnaires. Quality of life and household deprivation were also measured. T-tests compared means, and χ^2^ proportions; logistic regression examined predictors of ever having sex. 120(41%) PHIV and 31(32%) HIV- young people were male, 254(86%) and 70(73%) were black, median age 16 [IQR 15,18] and 16 [14,18] years respectively. 77(26%) PHIV had a previous AIDS diagnosis. 93(32%) PHIV and 38(40%) HIV- had ever had sex; median number of partners was 3 [1,6] and 4 [1,6] respectively. 54 (41%) of 131 young people who were sexually active reported not always using condoms, including 32% (30/93) of PHIV. In multivariable analysis, older age, male sex, worse deprivation score, worse quality of life, and alcohol and/or drugs were associated with ever having sex, but not HIV status. 12/30 PHIV reporting unprotected sex had at least one HIV viral load ≥200c/ml in the previous 12 months. Age at first sex and number of sexual partners were similar among PHIV and HIV-, and comparable to normative data. In conclusion, small numbers of PHIV reported condomless sex with a detectable viral load, which could result in HIV transmission, indicating the need for targeted sexual health and ART adherence interventions for young people with perinatal HIV.

## Introduction

Children with perinatal HIV are surviving to adolescence and adulthood and reaching developmental milestones. These include becoming sexually active [1–6], and transitioning to adolescent and adult clinics [7], the focus of which includes sexual and reproductive health. UK guidelines recommend that adults with HIV have regular sexual health assessments, access to high quality counselling and support to maintain good sexual health, and an annual offer of a full sexual health screen [8]. If not already immune, they should also undergo hepatitis B virus (HBV) vaccination.

Understanding sexual health and sexual risk behaviour in young people with perinatal HIV (PHIV) is important in order to tailor appropriate education on sexual health, including prevention of unintended pregnancy, acquisition of other sexually transmitted infections and the potential for onward transmission of HIV to partners and offspring [9]. However few studies have assessed factors associated with the initiation of sexual activity among PHIV young people. This is despite global estimates suggesting that just under two million adolescents aged 10–19 years are HIV positive, many of whom will have acquired HIV through mother to child transmission [10, 11].

Although dwarfed in terms of absolute numbers compared to sub-Saharan Africa, high income countries now have sizeable cohorts of PHIV young people reaching adulthood [12, 13]. To date, the larger studies of sexual behaviour in PHIV young people have been from the USA, and in younger adolescents [6, 9, 14, 15]. However little is known about sexual behaviour and sexual health needs in older adolescents, who are likely to engage in more sexual activity than younger adolescents, and where risk behaviours may differ by context, including in terms of family and social characteristics [4, 16]. Recruitment of appropriate HIV negative (HIV-) comparison groups from similar ethnic backgrounds and environments is critical to examine the effect of HIV status on sexual behaviour, as often the background characteristics of PHIV young people and their families in high income countries differ from the wider communities within which they live [17, 18].

The UK and Ireland has a relatively small number of people living with perinatal HIV compared to other countries globally, but a large proportion are now reaching adolescence and young adulthood [19, 20], and are facing the challenges of living with HIV while navigating this key developmental phase. The majority of the UK cohort is of sub-Saharan origin, and half were born outside the UK [21]. In a previous cross-sectional study of sexual and reproductive health outcomes of 52 PHIV young people attending a transition clinic in London [22], with a median age of 20 years, most (79%) were sexually active, and the median age at first sex was 16 years. In this study we explore the sexual health of a larger cohort of PHIV young people, as well as a comparable control group of adolescents affected by HIV, in England. We investigate prevalence and predictors of ever having sex, and ever having unprotected sex. We also described uptake of human papillomavirus (HPV) and HBV vaccination, and menarche, pregnancy and reproductive intentions of participants.

## Materials and methods

The Adolescents and Adults Living with Perinatal HIV (AALPHI) cohort is a prospective study evaluating the impact of HIV on young people with perinatal HIV-infected and HIV-affected but uninfected young people. Detailed methods have been reported elsewhere [23]. Briefly, PHIV young people were aged 13 to 21 years, had a history of paediatric care in the UK/Ireland, and were aware of their HIV status for at least 6 months [17, 24]. HIV- young people were aged 13 to 23 years, HIV negative on a point-of-care test at interview, and lived in the same household as a PHIV participant in AALPHI, or had a sibling, friend or partner who was a PHIV participant in AALPHI, or had an HIV positive parent.

Patient information Sheets (PIS) and consent forms were reviewed by staff and young people at two voluntary support groups to ensure the language and content were easily understood and age appropriate. Ethical approval was obtained from Leicester Research Ethics Committee (reference 12/EM/0012), who waived the requirement for parental consent for participants aged under 18 years where the participant demonstrated their own capacity to consent. This involved study research nurses having discussions with clinicians and/or voluntary sector staff about the young person’s physical and emotional state as well as their capacity to consent, prior to the young person being approached to participate in the study. Furthermore, to ensure young people had adequate information to decide whether to participate in the study, research nurses read the PIS to the young person and answered any queries. In addition, all the points in the consent form were discussed and questions posed throughout the consent process to help the research nurse decide if the young person had the ability and maturity to consent to participating in the study. Participants gave written informed consent, except where they lacked the capacity, in which case parents or guardians gave written informed consent and participants gave written assent.

Participants were enrolled between 2013 and 2015 and underwent a two hour face-to-face interview with a trained research nurse which included computer-assisted self-interview (CASI) questions about sexual health and onset of menarche. Sexual health questions included having ever had vaginal or anal sex, grouped age at first sex, vaginal or anal sex in the last 12 months, number of sexual partners, age at menarche and regularity of periods for girls, contraception methods, experience of pregnancies and their outcomes, sexually transmitted infections (STIs), history of HPV and HBV vaccination, and reasons for never having sex. Tanner stage (genitalia and pubic hair for males, and breasts and pubic hair for females) was based on self-report, with participants picking the picture which most represented their own stage of development [25]. It was then categorised into three groups (Tanner 1–2, Tanner 3–4, Tanner 5), similar to other published analyses.[26] BMI was categorised using WHO BMI z-scores (http://www.who.int/growthref/who2007_bmi_for_age/en/) as follows: <-2 standard deviations (SD) for underweight; ≥-2SD—<+1SD for normal weight; ≥+1SD—≤+2SD for overweight; >+2SD for obese. Neurocognitive Performance z-scores across 6 domains (NPZ-6) were calculated according to previously published methods [23], and the Income Deprivation Affecting Children Index (IDACI) was used to measure household deprivation, with higher scores indicating worse deprivation [27]. Alcohol use was measured using the Alcohol Use Disorders Identification Test (AUDIT) [28] and recreational drug questions were taken from the British Crime Survey [29]. Participants were asked if they were in education, employment, or not in education or employment, with questions being taken from the Avon Longitudinal Study of Parents And Children (http://www.bristol.ac.uk/alspac/), and were asked about the vital status of each biological parent. Quality of life was measured using the Pediatric Quality of Life Inventory (PedsQL), and scored according to manufacturer instructions using 25 unit steps [30]. Anxiety and depression were measured using the Hospital and Anxiety Scale (HADS) score [31] and the Rosenberg Self-Esteem Scale [32] was used to measure self-esteem. There were a small number of reports of actual harm from participants. In all instances young people reported that other health professionals were already helping with these matters, but permission was sought from the participant to confirm this with the clinician at their HIV clinic.

Data were analysed using STATA version 13 (Stata Corp, College Station, Texas, USA). T-tests compared means, and χ^2^ proportions. Medians were compared using the non-parametric Wilcoxon rank-sum test. The effect of potential predictors (listed in Table 1) on ever having sex (defined as vaginal and/or anal sex, also referred to in this paper as being “sexually active”), and ever having unprotected sex (using condoms never, sometimes or mostly, compared to always) among those who were sexually active, were explored using logistic regression and Wald p-values. Variables attaining a *p* value of less than 0.15 in univariable analyses were considered in multivariable analysis using backwards selection, as well as the *a priori* variables age and gender, and a two-tailed *p* value of less than 0.05 was considered statistically significant [33]. Due to small numbers of YPs reporting unprotected sex only the *a priori* variables and HIV status were included in the multivariable model for this outcome.

**Table 1:** Characteristics of PHIV and HIV- participants.

	*PHIV* (n = 296)	*HIV-* (n = 96)	*p value*
	*n (%) or median [IQR] or mean [SD]*		
**Age at interview**	16 [15, 18]	16 [14, 18]	0.74
**Male**	120 (41%)	31 (32%)	0.15
**Ethnicity (black)**	254 (86%)	70 (73%)	<0.01
**Born outside of UK/Ireland**	173 (58%)	46 (48%)	0.07
**Recruited in London**	210 (71%)	70 (73%)	0.71
**Death of one/ both parents**	101 (36%)	21 (23%)	0.02
**Occupation: In education**	273 (92%)	87 (91%)	0.21
**Not in education/ training**	15 (5%)	3 (3%)	
**Employed**	8 (3%)	6 (6%)	
**Lives with parents**	268 (91%)	85 (89%)	0.51
**IDACI deprivation score**	0.4 [0.2]	0.5 [0.1]	<0.01
**Ever alcohol**	122 (42%)	43 (46%)	0.50
**Ever smoking**	54 (19%)	24 (26%)	0.14
**Ever recreational drugs**	43 (15%)	26 (29%)	<0.01
NPZ-6 score*	-0.5 [0.9]	-0.3 [0.8]	0.03
Hospital Anxiety & Depression Scale anxiety score**	6.5 [3.9]	6.0 [4.1]	0.38
Hospital Anxiety & Depression Scale depression score**	3.9 [3.1]	3.6 [3.0]	0.40
PedsQL quality of life score***	75.8 [14.0]	79.7 [13.7]	0.02
Rosenberg self-esteem score****	20.3 [6.0]	20.3 [5.9]	0.83
**BMI for age z score**	0.7 [2.1]	0.5 [1.5]	0.67
**Male Tanner stage (self-reported)**	hhh jjj		
**1–2**	7 (7%)	2 (9%)	0.59
**3–4**	45 (48%)	8 (35%)	
**5**	43 (45%)	13 (56%)	
**Female Tanner stage (self-reported)**	444		
**1–2**	2 (1%)	0 (0%)	0.22
**3–4**	68 (47%)	18 (35%)	
**5**	74 (52%)	33 (65%)	
**Age at menarche (females only)**	13 [12, 14]	12 [11, 13]	0.03

* Summary z-score for cognitive performance across six domains [23]

** Scores are from 0–21; higher scores indicate more severe anxiety or depression [31]

*** Scores are from 0 to 100; higher scores indicate better quality of life [30]

****Scores are from 0–30; higher scores indicate better self-esteem

## Results

### Characteristics of PHIV and HIV- young people

A total of 296 PHIV and 96 HIV- young people contributed sexual health data. Of the 96 HIV- young people, 38 (40%) were siblings of PHIV young person in the study, 7 (7%) had PHIV siblings who were not in the study, 47 (49%) had a HIV positive mother, and 4 (4%) had a close friend who was a PHIV young people. Around a third in each group were male, median age was 16 years, the majority were black African, and around half were born abroad (Table 1). The majority of participants were at school and lived with their parents. For 36% of PHIV and 23% of HIV- young people one or both parents had died. Nearly half had ever had alcohol, a quarter had ever smoked, and 15% of PHIV and 29% of HIV- young people reported ever having taken recreational drugs. Mean NPZ-6 and quality of life scores were lower in the PHIV group (p = 0.03, p = 0.02 respectively), but there was no difference in anxiety, depression or self-esteem scores between PHIV and HIV- young people (all p>0.2). For males, less than 10% each of PHIV and HIV- young people self-reported Tanner stage 1–2, and for females, only two PHIV and no HIV- young people self-reported Tanner stage 1–2. Forty-five per cent and 52% of PHIV males and females reported stage 5, compared to 56% and 65% of HIV- males and females.

### HPV and HBV vaccination

Overall self-reported prevalence of HPV vaccination was 6% (7/118) in PHIV males, 0% (0/31) in HIV- males, 66% (116/176) in PHIV females, and 58% (38/65) in HIV- females. For HBV, 29% (34/117) PHIV males, 10% (3/30) HIV- males, 32% (57/176) PHIV females and 6% (4/65) HIV- females reported having completed or were currently completing the full course of vaccinations.

### Sexual behaviour

A total of 32% of PHIV and 40% of HIV- young people reported ever having vaginal or anal sex (p = 0.23, Table 2), of whom the proportion who had sex before age 15 years was 20% and 19% respectively, with no differences by gender (males 36% vs females 28% overall, p = 0.13). Thirty per cent of PHIV and 39% of HIV- young people had ever had vaginal sex (p = 0.21), of whom 20% and 19% respectively had vaginal sex before age 15 years (p = 0.51). Seven per cent of PHIV males and 10% of HIV- males, and 5% of PHIV females and 5% of HIV- females, had ever had anal sex. Of these, one PHIV male and one HIV- female had anal sex before age 15 years. Two per cent (2/119) of PHIV males, no (0/31) HIV- males, 1% (2/176) of PHIV females, and 2% (1/65) of HIV- females, had ever had anal sex but not vaginal sex.

**Table 2:** Sexual behaviour of PHIV and HIV- participants.

	PHIV			HIV-			χ^2^ p*
	Male (n = 119)	Female (n = 176)	Total (n = 295)	Male (n = 31)	Female (n = 65)	Total (n = 96)	
	n (%) or median[IQR]						
**Ever had vaginal or anal sex**	43 (36%)	50 (28%)	93 (32%)	14 (45%)	24 (37%)	38 (40%)	0.23
**Ever had vaginal sex**	41 (34%)	48 (27%)	89 (30%)	14 (45%)	23 (35%)	37 (39%)	0.21
**Age first had vaginal sex**							
≤14 years	11 (28%)	6 (13%)	17 (20%)	2 (14%)	5 (22%)	7 (19%)	0.51
15–19 years	27 (69%)	40 (83%)	67 (77%)	12 (86%)	18 (78%)	30 (81%)	
≥20 years	1 (3%)	2 (4%)	3 (3%)	0 (%)	0 (0%)	0 (0%)	
**Vaginal sex in last 12 months**	36 (30%)	46 (26%)	82 (28%)	13 (42%)	22 (34%)	35 (36%)	0.23
**Ever had anal sex**	8 (7%)	9 (5%)	17 (6%)	3 (10%)	3 (5%)	6 (6%)	0.49
**Age first had anal sex**							
≤14 years	1 (13%)	0 (0%)	1 (6%)	0 (0%)	1 (33%)	1 (17%)	0.62
15–19 years	7 (88%)	8 (89%)	15 (88%)	3 (100%)	2 (67%)	5 (83%)	
≥20 years	0 (0%)	1 (11%)	1 (6%)	0 (0%)	0 (0%)	0 (0%)	
**Anal sex in last 12 months**	5 (4%)	8 (5%)	13 (4%)	3 (10%)	3 (5%)	6 (6%)	0.53

*Comparing total PHIV to total HIV-

Prevalence of vaginal and/ or anal sex increased with increasing age at interview, from 6% (9/154) and 1% (2/153) among those aged <16 years to 49% (117/237) and 9% (21/237) among those aged ≥16 years respectively (p<0.001, p = 0.001 respectively). Of those who were sexually active, males had a median of five sexual partners since they were first sexually active (interquartile range (IQR) 1, 10), compared to two for females (1, 5) (median test p = 0.009), although there was no difference by HIV status (PHIV 3 (1, 6), HIV- 4 (1, 6), p = 0.60).

### Menarche, contraception and pregnancy among females, and reproductive intentions

Median age at interview for female PHIV young people was 16 years (interquartile range (IQR) 14, 18), and 96% (168/175) reported having started menstruation, with median age at menarche of 13 years (IQR 12, 14). Of the 7 PHIV young women who had not menstruated, median age was 14 years (13, 20), and two had a previous CDC C event. Median age at interview of HIV- females was 16 years (IQR 14, 18), and 92% (60/65) had started menstruation, with median age at menarche of 12 years (IQR 11, 13). Eleven per cent (19/168) of PHIV females and 18% (11/60) of HIV- females reported that their periods were irregular; median age for those reporting irregular periods was similar to those reporting regular periods (16 [14–17] and 16 [15–18] respectively, p = 0.23)

Of those with irregular periods, 0%, 69%, 15% and 15% were underweight, normal weight, overweight and obese respectively, and similarly 2%, 57%, 24% and 18% for regular periods. Sixteen per cent (3/19) of PHIV and 27% (3/11) of HIV- females reporting irregular periods had used the long-term contraception methods in the last 12 months, and 16% (3/19) and 36% (4/11) have ever used a depo Provera injection method of contraception respectively.

Two-thirds of PHIV and a third of HIV- participants who were sexually active reported always using condoms, 17% and 21% mostly, and 15% and 42% sometimes or never (Table 3). Of those who did not always use condoms, the majority reported having unprotected vaginal sex in the last 12 months, and the median number of sexual acts without a condom was two (IQR 1, 4) for PHIV and four (2, 20) for HIV- young people. Other forms of contraception which both females and males who were sexually active and the people they had sex with had used included the oral contraceptive pill, implant, depo Provera injections, intrauterine device, intrauterine system, and a patch (Table 3).

**Table 3:** Contraception use of PHIV and HIV- participants who were sexually active.

	*PHIV* (n = 93)	*HIV-* (n = 38)
	*n (%) or median [IQR] or mean [SD]*	
**Use of condoms**		
Always	63 (68%)	14 (37%)
Mostly	16 (17%)	8 (21%)
Sometimes or never	14 (15%)	16 (42%)
**Other forms of contraception used**		
Oral contraceptive pill	18 (20%)	12 (32%)
Implant	11 (12%)	6 (16%)
Depo Provera injection	12 (13%)	5 (14%)
Intrauterine device	6 (7%)	3 (8%)
Intrauterine system	5 (6%)	1 (3%)
Patch	3 (3%)	1 (3%)

Six PHIV and five HIV- female participants reported ever having been pregnant, none at the time of interview. One had been pregnant three times, and three had been pregnant twice; 2 were PHIV and 2 were HIV- young people. No participant reported a live birth. Two participants reported having terminations, one for two pregnancies and the other one of their two pregnancies (1 unknown). Eight reported a total of 10 miscarriages, and for one participant the outcome of the 2 pregnancies was unknown. Only two participants, both PHIV, reported that they were planning to become pregnant in the next year, and were aged 16 and 19 years. Seven PHIV and six HIV- participants reported ever having an STI, of whom four were male and nine female. In the last 12 months, two reported having genital herpes, 8 chlamydia and gonorrhoea. Four had bacterial vaginosis. Of the 9 female participants reporting ever having an STI, three reported ever being pregnant, compared to 13% (8/63) in those female participants who have never had an STI. Of those who reported being sexually active, 42% (41/97) of PHIV and 13% (5/38) of HIV- were aware of post-exposure prophylaxis (PEP). Five PHIV participants reported that a partner had taken PEP after unprotected sex.

### Factors associated with ever having sex

Before and after adjustment for other factors, there was no difference in the odds of ever having sex for PHIV compared to HIV- young people (adjusted odds ratio (aOR) = 0.95 (95% CI 0.44, 2.05), p = 0.89, Table 4). However, odds increased with increasing age (aOR = 1.46 (1.26, 1.68) per year increase, p<0.001), with 41% of 16–18 year olds and 66% of ≥19 year olds ever having sex. It was also higher in those who had a worse deprivation score (aOR = 1.20 (1.01, 1.42) per 0.1% increase, p = 0.041), and those who had ever had alcohol (aOR = 2.85 (1.46, 5.58), p = 0.002) or recreational drugs (aOR = 3.39 (1.53, 7.51), p = 0.003). Prevalence was also lower among females compared to males (aOR = 0.39 (0.20, 0.76), p = 0.006), and lower among those with a better quality of life (aOR = 0.56 (0.32, 0.99) per 25 points increase, p = 0.046).

**Table 4:** Prevalence, and univariable and multivariable predictors of ever having vaginal and/or anal sex.

Variable	Category	Ever sex		Univariable			Multivariable		
		n/total	%	OR	95% CI	p	aOR	95% CI	p
HIV status	HIV-	38/96	40%	1	-		1	-	
	HIV+	93/295	32%	0.68	0.42, 1.10	0.119	0.95	0.44, 2.05	0.894
***Sociodemographics***									
Age group at interview	≤15 years	10/154	7%	-	-		-	-	
	16–18 years	63/153	41%	-	-		-	-	
	≥19 years	21/32	66%	-	-		-	-	
Age	Per year increase	-	-	1.72	1.53, 1.94	<0.001	1.46	1.26, 1.68	<0.001
Gender	Male	57/150	38%	1	-		1	-	
	Female	74/241	31%	0.69	0.45, 1.08	0.092	0.39	0.20, 0.76	0.006
Location of enrolment	London	101/279	36%	1	-				
	Elsewhere	30/112	27%	0.65	0.40, 1.05	0.081			
Occupation (*v*. in education)	In education/training/employment	120/373	32%	1	-				
	Not in education/training/employment	11/18	61%	3.16	1.19–8.35	0.021			
***Social***									
Who participant lives with	Parents	100/352	28%	0.09	0.04, 0.22	<0.001			
	Other	31/38	82%	1	-				
Income Deprivation Affecting	< sample median	55/168	33%	-	-		-	-	
Children Index (IDACI) score	≥ sample median	51/167	31%	-	-		-	-	
	Per 0.1% increase (worse)			1.12	0.98, 1.28	0.093	1.20	1.01, 1.42	0.041
**Lifestyle**									
Alcohol use	Never	37/220	17%	1	-		1	-	
	Ever	91/165	55%	6.27	3.91, 10.07	<0.001	2.85	1.46, 5.58	0.002
Smoking	Never	85/307	28%	1	-				
	Ever	43/77	56%	3.23	1.93, 5.43	<0.001			
Recreational drug use	Never	76/306	25%	1	-		1	-	
	Ever	51/69	74%	8.20	4.51, 14.90	<0.001	3.39	1.53, 7.51	0.003
**Neurocognitive/ quality of life**									
PedsQL (quality of life)	≥70	84/268	31%	-	-		-	-	
	<70	46/116	40%	-	-		-	-	
	Per 25 points increase (better)			0.64	0.44, 0.94	0.024	0.56	0.32, 0.99	0.046
Neurocognitive Performance	≥-1	97/275	35%	-	-				
Z-score (NPZ-6)	<-1	23/86	27%	-	-				
	Per 1 point increase			1.38	1.03, 1.83	0.029			

Notes

OR, odds ratio; aOR, adjusted odds ratio, A priori variables (age and gender), and those with univariable *p*<0.15 or multivariable *p*<0.05 are presented here.

### Factors associated with ever having unprotected sex

Thirty (32%) of 92 PHIV and 24 (63%) of 38 HIV- who were sexually active reported not always using condoms (p = 0.001). The proportion reporting unprotected sex was similar by gender (39% of males, 43% of females, p = 0.59) and median age of those having unprotected sex was similar to those not reporting unprotected sex (18 (IQR 17, 19) and 18 (17,20) respectively, p = 0.50). In a multivariable model including those who were sexually active, after adjustment for *a priori* variables age and gender (both p>0.2), HIV status was predictor of ever having unprotected sex: HIV- young people were more likely to report having unprotected sex than PHIV young people (aOR = 3.80 [95% CI 1.69–8.55], p = 0.001.

Of the 30 PHIV who reported not always using condoms, 12 (40%) had at least one viral load ≥200c/ml in the 12 months prior to interview (of whom 4 were 1,000–9,999c/ml, 5 were 10,000–99,999, and 3 were ≥100,000c/ml).

### Reasons for not having sex

Of the 248 who had not had vaginal or anal sex, 22% cited religious beliefs, 29% fear of HIV transmission, 67% not being ready yet, 6% family pressure, 2% partner didn’t want to, and 18% not having had the opportunity, as reasons. The median age of those never having sex was 15 years (IQR 14, 17) at interview compared 18 years (17, 20) for those who were sexually active (p<0.001).

## Discussion

To our knowledge, ours is the first quantitative study outside of the USA describing sexual behaviour in PHIV with a comparison group of HIV- young people (aged 13–23). Our participants had a median age of 16 years and many were young adults aged ≥18 years at the time of interview, although most remained at school and resided with their parents.

Just under three quarters of PHIV and HIV- girls self-reported that they had received HPV vaccination. In the UK, HPV vaccination has been available since 2008 free of charge to all girls, administered within the school vaccination program at the age of 12/13 years. The national uptake of two doses of the Human Papillomavirus 9-valent vaccine recombinant, which also prevents genital warts, is around 80% [34]. Boys are not eligible for the routine vaccination program, although HPV vaccination is recommended for both sexes in the context of HIV in national guidance [35]. The low prevalence of HPV vaccination in HIV infected boys (<10%) highlights the disparity between guidance and delivery outside a national vaccination program and without funding. Results suggest levels in girls are relatively high considering that some female young people may have either forgotten about a previous vaccination or have been to old to qualify for the current programme.

Despite World Health Organization recommendations for universal infant HBV vaccination in 1992, it was not until August 2017 that HBV vaccination was incorporated into the routine infant UK schedule, with previous HBV vaccination offered free of charge only to perceived high risk groups. Whilst this may explain the low rates of HBV vaccination in the HIV negative group in our study, all children living with HIV should receive HBV vaccination by early adolescence [35] and prevalence of self-reported vaccination are worryingly low. Previous data suggests HBV vaccination in adolescents living with HIV occurs infrequently prior to transition to adult care [22] and requires considerable improvement in paediatric services, as well as careful assessment of status on presentation to adult care [36]. Thus results from our study suggest the need for review of HPV and HBV vaccination status at the point of transition to adult care.

Around a third of participants in our study reported vaginal and/or anal sex, with no difference in incidence by HIV status. In contrast, a study of 420 young people with mean age 15 years in New York City, of whom 39% had perinatal HIV, PHIV young people were significantly less likely to engage in penetrative sex than HIV- young people [14]. However in that study, the HIV- group were a mix of HIV exposed and HIV unexposed young people, and the research was conducted over 10 years ago and with very different contextual factors. Another study, providing general population estimates, is a large birth cohort in south-west England (Avon Longitudinal Study of Parents and Children (ALSPAC)), which found that at a mean age of 18 years (two years older than in our study), 39% of 1,244 males and 61% of 1,633 females reported ever having sex, giving an overall prevalence of 51% [37]. In our study 41% of 16–18 year olds and 66% of ≥19 year olds had ever had sex, remarkably similar to ALSPAC. Participants in ALSPAC were all born in England, and more likely to have higher educational attainment and white ethnicity, and less likely to be eligible for free school meals, compared to national data, and likely our sample [38], highlighting the importance of having both PHIV and HIV- young people from similar backgrounds as comparators.

Age at first sex was similar between PHIV and HIV- participants, concurring with other studies suggesting no effect of HIV status on age at sexual debut [9, 14, 15]. The majority of our participants were aged 15 to 19 years at first sex, similar to data from the National Surveys of Sexual Attitudes and Lifestyles (Natsal) in Britain, which reported a median age of 16 years at first heterosexual intercourse for both men and women [22]. In our study, 6% of all participants reported having vaginal or anal sex before the age of 15 years, whilst in Natsal 31% of men and 29% of women aged 16–24 years reported heterosexual intercourse before age 16 years, much of which may occur at age 15 years and so difficult to compare to our data [39]. The median age at transfer to adult care across the national cohort of PHIV in the UK and Ireland was 17 years [19], with many participants becoming sexually active whilst still formally in “paediatric” care, although many paediatric clinics now have a young people focus including evening clinics.

Factors associated with increased odds of sexual initiation in our analysis were older age, male sex, worse deprivation score, poorer quality of life, and alcohol and/ or recreational drug use. Thirty-eight per cent of males and 31% of females reported ever having sex, and after adjustment for age, males had higher odds of ever having sex than females. The association between worse deprivation score, and also poorer quality of life, and higher odds of ever sex highlights the important role of social factors on sexual behaviour. The positive associations between alcohol and/or drug use and increased sexual risk behaviour have been previously described in adolescent populations, both HIV infected and the uninfected peers [4, 40–45].

In terms of condom use, nearly two-thirds of HIV- participants and a third of PHIV who were sexually active reported not always using condoms, and the majority of these young people reported unprotected vaginal sex in the last 12 months. Furthermore, forty per cent of PHIV who reported inconsistent condom use had viral load ≥200c/ml in the 12 months prior to interview. Taken together, these findings indicate risk behaviour in a small proportion of PHIV in our study, exposing sexual partners to the risk of HIV transmission. Comparisons to other studies of PHIV are limited due to most being conducted prior to the PARTNER study which showed an absence of within-couple HIV transmission events in discordant couples practising condomless sex where the HIV positive partner was established on suppressive ART (VL <200c/ml); these older studies did not distinguish between unprotected sex with and without suppressed viral load [46]. In the New York City study, 65% of sexually active PHIV and 50% of perinatally HIV-exposed, uninfected young people reported unprotected sex, and a third of participants reported recent non-adherence, of whom 45% had detectable HIV RNA levels [47]. Another study of 330 PHIV in the USA, with mean age 13.5 years, reported that 62% of those who were sexually active had engaged in unprotected sexual intercourse, and 42% of the sexually active participants had a viral load ≥5,000c/ml [6]. Related to the issue of unprotected sex, 11 participants reported ever being pregnant, no pregnancies resulted in a live birth, and many resulted in miscarriages, highlighting the importance of ongoing conversations with young people in clinic about their reproductive health.

Our study has several limitations. Firstly, sexual behaviour and HBV and HPV vaccine uptake data were based on self-report, and not validated through review of medical records, so could be subject to recall bias. We attempted to mitigate biases in sexual behaviour reporting through the use of confidential computer based interviewing [48]. Furthermore HPV vaccine is given in schools and so would not be recorded in medical records. Secondly, we also asked only very simple questions about condom use. Results from a study of 135 young people engaging in vaginal sex at mean age 19 years reported how adding specific questions about condom use from the start to the finish of the sexual encounter doubled the prevalence of unprotected sex [5], and so our results may be considered a minimum estimate. Thirdly, most of our participants were recruited from HIV outpatient departments in hospitals, and we may not have reached those who were not retained in care and who may have had higher risk behaviour. However we also recruited from the voluntary sector, and a comparison of the demographic characteristics of PHIV included in our study compared to the wider cohort of adolescents with PHIV in the UK and Ireland suggested no major differences [23]. Fourthly, we only asked about reproductive intentions over the subsequent year, whilst a longer time frame may have elicited important information on reproductive desires among this group.

In conclusion, our results suggest no difference between levels of sexual behaviour in PHIV and comparable HIV uninfected young people. Levels were also similar to national data, and age at first sex in our study was also similar to national data. However a minority of PHIV participants engaged in condomless sex with a detectable viral load, which could result in HIV transmission, indicating the continued need for ongoing sexual health and ART adherence interventions. Future research could examine the impact of “Undetectable = Untransmissible” (https://www.preventionaccess.org/) [46] on sexual behaviour, condom use and adherence to ART in this unique population as they age into the third decade of life.
